# Both inflammatory and regulatory cytokine responses to malaria are blunted with increasing age in highly exposed children

**DOI:** 10.1186/s12936-017-2148-6

**Published:** 2017-12-29

**Authors:** Lila Farrington, Hilary Vance, John Rek, Mary Prahl, Prasanna Jagannathan, Agaba Katureebe, Emmanuel Arinaitwe, Moses R. Kamya, Grant Dorsey, Margaret E. Feeney

**Affiliations:** 1 Department of Medicine, University of California San Francisco, San Francisco, CA USA; 2 Department of Medicine, Stanford University, Stanford, CA USA; 3 Infectious Diseases Research Collaboration, Kampala, Uganda; 4 Makerere University College of Health Sciences, Kampala, Uganda; 5 Department of Pediatrics, University of California San Francisco, San Francisco, CA USA

**Keywords:** Cytokines, Malaria, Tolerance, Immunity, Age

## Abstract

**Background:**

Young children are at greatest risk for malaria-associated morbidity and mortality. The immune response of young children differs in fundamental ways from that of adults, and these differences likely contribute to the increased susceptibility of children to severe malaria and to their delayed development of immunity. Elevated levels of pro-inflammatory cytokines and chemokines in the peripheral blood during acute infection contribute to the control of parasitaemia, but are also responsible for much of the immunopathology seen during symptomatic disease. Clinical immunity to malaria may depend upon the ability to regulate these pro-inflammatory responses, possibly through mechanisms of immunologic tolerance. In order to explore the effect of age on the immune response to malaria and the development of clinical immunity, cytokines and chemokines were measured in the plasma of children at day 0 of an acute malaria episode and during convalescence.

**Results:**

Younger children presenting with acute malaria exhibited much higher levels of TNF, IL2, and IL6, as well as increased Th1 associated chemokines IP10, MIG, and MCP1, compared to older children with acute malaria. Additionally, the regulatory cytokines IL10 and TNFRI were dramatically elevated in younger children compared to older children during acute infection, indicating that regulatory as well as pro-inflammatory cytokine responses are dampened in later childhood.

**Conclusions:**

Together these data suggest that there is a profound blunting of the cytokine and chemokine response to malaria among older children residing in endemic settings, which may be due to repeated malaria exposure, intrinsic age-based differences in the immune response, or both.

## Background

*Plasmodium falciparum* malaria is responsible for one in every five childhood deaths in Africa [1]. The risk for severe disease and death is highest in early childhood, and declines with increasing age as clinical immunity develops after repeated exposures. Children living in malaria-endemic areas eventually develop clinical immunity to malaria, which protects against symptoms but not against infection. However, this immunity develops only after years of sustained exposure. The reasons for this delayed immunity are probably multifactorial. Antigenic diversity of the parasite likely plays a role, and this diversity comes in two forms: genetically stable allelic polymorphisms of antigen-coding genes, which vary between isolates, and antigenic switching by individual isolates, which alters antibody-binding sites of malarial proteins expressed on the surface of infected red blood cells [2]. However, evidence also suggests that age-related differences in the developing immune system of children contribute to their slow development of immunity [3, 4].

Compared to adults, infant immune cells (including monocytes, dendritic cells and T cells) are biased toward the production of regulatory, Th2, and Th17 cytokines and exhibit lower production of proinflammatory Th1 cytokines [5–11]. As a result, infants are more susceptible than adults to numerous viral, bacterial, and protozoal pathogens, and generally exhibit delayed pathogen clearance compared to older children and adults. Hence, it is not surprising that the acquisition of anti-malarial immunity follows a different trajectory in young children than in adults, as has been described in several longitudinal studies of Javanese transmigrants [3, 4]. These studies suggest that adults are initially more susceptible to severe malaria than young children, but develop immunity to repeated infection much more rapidly.

During an acute malaria infection, cytokines and chemokines are elevated in peripheral blood and contribute to parasite clearance, but are also likely to be responsible for many of the symptoms and pathological changes seen during malaria disease. Experimental data indicate that the balance of pro and anti-inflammatory signals plays a large role in dictating the outcome of an infection, i.e. whether it leads to protection and/or immunopathology [12]. Accordingly, clinical immunity might also depend upon the ability to moderate pro-inflammatory responses, as suggested by recent data from our group and others [13–15]. Indeed, malaria-naïve adults experimentally infected with *P. falciparum* can be divided into two prognostic groups based on their plasma cytokine profiles [16]. The first group is characterized by a strong inflammatory response (defined as an eight to 82-fold increases in IFNγ above baseline, with similarly significant increases in TNF, IL12p70 and the chemokine MIG/CXCL9) and exhibits rapid parasite control but more severe symptoms. The second group is characterized by early TGFβ production and a weaker inflammatory response (less than a twofold increase in IFNγ above baseline), and exhibits weaker parasite control and fewer symptoms. Few data are available regarding cytokine levels in children, or in residents of endemic areas who are repeatedly exposed to malaria, and hence little is known regarding how plasma cytokines relate to outcomes of malaria infection in these settings. Mshana et al. observed an inverse relationship between age and plasma IFNγ and TNF in Gabonese children some years ago [17], but these results have yet to be extended to a more comprehensive view of the immune response. As children are the population most at risk for malaria-associated morbidity and mortality, it would be helpful to understand how age impacts cytokine and chemokine biomarkers of infection, and how childhood immune responses fit into to the paradigm put forward by studies of malaria in previously naïve adults.

Here, the cytokine response to malaria in younger (1–3 years) and older (7–10 years) children was compared. An array of 20 cytokines and chemokines were measured in plasma at the time of presentation with malaria, and again 3 weeks after treatment. The resulting data indicate that younger children, similar to naïve and partially immune adults, are more likely to mount a strong pro-inflammatory response to malaria, but that this response is tempered by large amounts of regulatory cytokine production. The response of older children was comparatively dampened, suggesting age-related differences in immunity and the development of partial clinical tolerance.

## Methods

### Ethics approval

Written informed consent was obtained from the parent/guardian of all study participants. Study protocols were approved by the Uganda National Council of Science and Technology and the institutional review boards of the University of California, San Francisco, and Makerere University.

### Study participants

Samples were obtained from a sub-study of participants in the East African International Centres of Excellence in Malaria Research ‘PRISM’ Nagongera study cohort that was initiated in 2011 and is ongoing. This cohort consists of 100 households within the Nagongera sub-county in Tororo district. Details of this study have been published elsewhere [18]. Malaria transmission in this region is holoendemic, with an annual EIR of 310 infective bites per person year. For the work presented here, a subset of children presenting with acute febrile malaria were selected to undergo an initial blood draw and follow up draws at 3 weeks after beginning treatment. Subjects were selected using the following criteria: (1) duration of fever ≤ 48 h, (2) *P. falciparum* asexual parasite density > 5000/μl, (3) absence of complicated malaria, (4) no clinical suspicion of non-malarial intercurrent illness (e.g. no upper respiratory symptoms or diarrhoea), (5) no treatment for malaria in the prior month. Subjects were treated for malaria with artemether-lumefantrine, per protocol. In total, plasma samples from 48 children were analysed: 25 from children 1–3 years old and 23 from children 7–10 years old. The study cohort characteristics are presented in Table 1.

**Table 1:** Clinical characteristics

	1–3 year olds (n = 25)	7–9 year olds (n = 23)	P value Wilcoxon rank
Malaria incidence in the preceding 365 days, median episodes ppy (IQR)	7 (5–8.29)	6 (4–6)	ns
Malaria incidence in the following 365 days, median episodes ppy (IQR)	5.82 (3.35–7)	1.44 (0–4)	0.0008
Parasite density at day 0, median parasites/μl (IQR)	39,720 (24,960-66,800)	17,880 (11,080-28,960)	0.0002
Haemoglobin at day 0, median g/dl (IQR)	10.8 (10.2–11.5)	11.3 (10.4–12.1)	0.03

*IQR* interquartile range, *ppy* per person year

### Parasite densities/haemoglobin

Thick blood smears were stained with 2% Giemsa for 30 min. Thick smears were evaluated for the presence of parasitaemia (asexual forms only) and gametocytes. Parasite densities were calculated by counting the number of asexual parasites per 200 leukocytes (or per 500, if the count was less than 10 parasites or gametocytes per 200 leukocytes), assuming a leukocyte count of 8000/μl. A smear was considered negative after reviewing 100 high-powered fields. For quality control, a second microscopist read all slides and a third reviewer was used to settle any discrepant readings. Haemoglobin concentration was assessed using a battery-operated portable haemoglobinometer (HemoCue Ltd., Angelholm, Sweden) and estimated to an accuracy of 1 g/dl.

### Sample processing

6–10 ml of blood were obtained in acid citrate dextrose tubes. Plasma was collected by centrifugation and cryopreserved in − 80 freezers. Samples were shipped to San Francisco on dry ice prior to thaw and analysis.

### Luminex

The concentrations of 20 cytokines and chemokines were measured in plasma samples using Luminex technology (Luminex Corporation, Austin, TX, USA). Fourteen cytokines [TNF, TNFRI, IFNγ, IL1β, IL2, IL4, IL5, IL6, IL7, IL8, IL10, IL12p70, IL17, IL23] and six chemokines [MIG, MDC, MIP1α, MCP1, IP10, TARC] were analysed using a custom R&D Magnetic Luminex Screening Assay Human Premixed Multi-Analyte Kit (Catalog Number LXSAHM). Plasma was diluted 1:2 as per the manufacturers recommendations. All samples were analysed in duplicate. Protocols were performed as indicated by the vendor: Briefly, 50 μl of provided standard or test sample, along with 50 μl of the mixed microparticles, were added to each well of a 96-well micro-titer plate. After 2 h of incubation and washing at room temperature, 50 μl of diluted biotin antibody cocktail was added to each well. Samples were incubated for another hour. After washing, 50 μl of diluted Streptavidin-PE were added to each well. The plate was incubated for 30 min, washed, and the microparticles were resuspended in 100 μl of wash buffer. The plate was read on a Luminex MAGPIX CCD Imager and data were analysed using xPONENT software (v4.2). A minimum of 50 beads per region were analysed. A curve fit was applied to each standard curve according to the manufacturer’s instructions and sample concentrations were interpolated from the standard curves. MFIs were background subtracted. Variability between duplicates was minimal, with an average coefficient of variance of 5.2%. Age groups and time points were randomly distributed between plates to minimize batch effects. The limit of detection for each analyte was as follows: TNF: 14.83 pg/ml, TNFR1: 220.58 pg/ml, IFNγ: 7.55 pg/ml, IL1B: 17.75 pg/ml, IL2: 30.62 pg/ml, IL4: 18.26, IL5: 17.57 pg/ml, IL6: 14.03 pg/ml, IL7: 5.84, IL8: 8.13 pg/ml, IL10: 13.64 pg/ml, IL12p70: 278.45 pg/ml, IL17a: 21.42 pg/ml, IL23: 239.36, MIG: 396.06 pg/ml, MDC: 59.01 pg/ml, MIP1α: 60.39 pg/ml, MCP1: 32.70 pg/ml, IP10: 3.78 pg/ml, TARC: 9.06 pg/ml. Samples below the limit of detection were either given a value at the limit of detection, or half the limit of detection when log-transformed for statistical analysis.

### Elisa

Human IFNγ BD OptEIA ELISA II Kits (Catalog # 550612) were used to quantify IFNγ levels in plasma. Samples were diluted 1:2 and run in duplicate using the protocol provided by the manufacturer. Plates were read on a SpectraMax M2 (Molecular Devices) and analysed using SoftMax Pro (v) software to fit a curve to the standards supplied by the vendor. Background absorbance was subtracted from sample measurements. The limit of detection for this assay was interpolated from the standard curve to be 5 pg/ml.

### Statistical methods

All statistical analyses were performed using Prism 7.0a (GraphPad) or STATA SE version 13.1 (College Station). Comparisons of malaria between age groups, as well as comparisons of cytokine and chemokine concentrations between age groups and timepoints, were done using the Wilcoxon rank sum test or the Wilcoxon signed-rank test for paired data (i.e. day 0 vs. week 3). Associations between parasite densities and cytokine/chemokine concentrations were assessed using Spearman correlation. All multivariate statistics were derived by linear regression models with transformed variables following log or square root-transformation of any non-normal variables. Two-sided P values were calculated for all test statistics and *P* < 0.05 was considered significant.

## Results

### Study design and clinical characteristics

Children who presented at the time of an acute malaria episode were enrolled to undergo an initial blood draw and a follow up draw 3 weeks after treatment. Subjects were recruited as part of a nested sub-study within an ongoing longitudinal cohort study of childhood malaria in Eastern Uganda. Details of this study have been published elsewhere [18]. The study site is notable for its extremely high malaria transmission intensity, with an annual entomological inoculation rate of 310 infective bites per person year as measured in 2013. For children in this sub-study (n = 48), data regarding malaria incidence, parasite density, and haemoglobin at the time of blood sampling are shown in Table 1. Incident episodes of malaria were defined as all febrile episodes accompanied by any parasitaemia, but not preceded by another treatment in the prior 14 days. To minimize heterogeneity in plasma cytokine levels resulting from incident malaria, children were excluded if they arrived to the clinic more than 48 h after the onset of fever, had a parasite density less than 5000 parasites/μl, had received anti-malarial treatment any time in the prior month, or if there was any clinical suspicion of non-malarial intercurrent illness (e.g. upper respiratory symptoms or diarrhoea).

Parasite density was assessed by blood smears performed on-site. Subjects were removed from the study if convalescent samples tested positive for parasitaemia by blood smear at 3 weeks after treatment. As expected, the median parasite density at day 0 for children in the 1–3 year age group was significantly higher than for children in the 7–10 year age group (Table 1). Haemoglobin levels in the peripheral blood were lower in younger children than older children (Table 1), but were not associated with parasitaemia levels in either age group.

### Pro-inflammatory cytokines are higher in young children with acute malaria

**Fig. 1:**
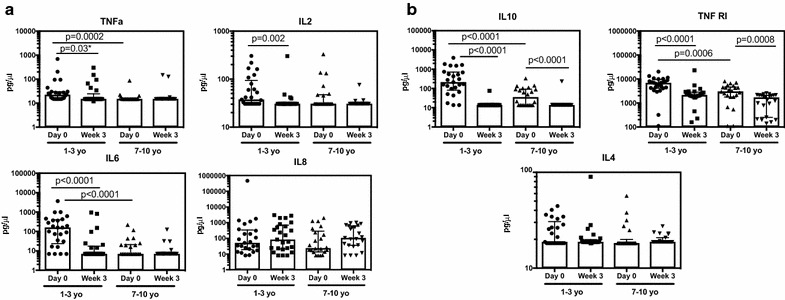
Plasma inflammatory (**a**) and regulatory (**b**) cytokine responses to malaria across age groups. Data are presented as scatter plots across age groups and timepoints. Boxes represent medians and whiskers represent the IQR of each group. Significant differences across age groups were assessed by Wilcoxon rank sum and the Wilcoxon sign-rank test was used for pairwise comparisons between timepoints. Only P values < 0.05 are shown. *Denotes p values that are not statistically significant (P > 0.0125) after Bonferroni multiple comparisons testing. Samples below the limit of detection are shown at the lower limit of detection. Only cytokines for which > 7% of samples were above the limit of detection appear in the figure

To assess the relationship between age and cytokine production during acute malaria, plasma was collected at presentation with acute malaria (during the first 48 h of symptoms) and 3 weeks after treatment. Plasma cytokine levels were measured using a multiplex bead array on a Luminex platform. Pro-inflammatory cytokines, including TNF and IL6, were significantly higher in the younger children than in older children at presentation (Fig. 1a). Indeed, only in the younger age group were TNF, IL6, and IL2 concentrations elevated during acute malaria (day 0) as compared to convalescence (week 3). IL8, while present at detectable levels in many children, did not differ by age or time-point.

Because parasite density also varied with age, the relationship between cytokine concentrations and parasite density was examined, both independently and as a covariate with age (Table 2). A positive relationship between parasite density and plasma TNF concentration has been noted previously [19]. Both age and parasite density were significant predictors of these cytokine concentrations in univariate analysis. However, in multivariate analysis, only age was the significant predictor for both cytokines, although the relatively small sample size may have limited the power of this analysis to detect a small influence of parasitaemia. The interaction between age and parasite density was not significant in either case. Further, stratifying by age group did not reveal any relationship between parasite density and IL6 or TNF concentrations at day 0. Thus it appears that age, but not parasite density, influences concentrations of the pro-inflammatory cytokines IL6 and TNF.

**Table 2:** Relationships to age and parasite density

Cytokine/ chemokine	Univariate			Multivariate analysis					1–3 yr olds		7–9 yr olds	
	Age group^a^	Parasite density		Age group		Parasite density		PD X AG	Parasite density		Parasite density	
	P	P	Coef	P	Coef	P	Coef	P	P	Coef	P	Coef
IL10	*–*	*0.0003*	0.50	*0.002*	− 0.72	0.2	0.45	*0.03*	*0.02*	0.46	0.6	− 0.13
TNFR1	*0.0006*	*0.006*	0.40	*0.02*	− 20.4	0.3	11.9	*0.03*	*0.03*	0.45	0.2	− 0.28
TNF	*0.0002*	*0.004*	0.42	*0.01*	− 0.35	0.9	0.12	0.8	0.6	0.11	0.4	0.20
IL2	0.1	0.1	0.22	0.6	− 0.05	0.3	0.15	0.2	0.4	0.19	0.6	− 0.12
IL4	0.1	0.3	0.16	0.5	− 0.04	0.5	0.04	0.7	0.6	− 0.12	0.5	0.16
IL6	*<* *0.0001*	*0.0007*	0.48	*0.001*	− 0.75	0.2	0.36	0.9	0.5	0.14	0.1	0.34
IP10	0.005	0.05	0.28	0.02	− 0.37	0.8	0.05	0.1	0.2	0.25	0.3	− 0.22
MIG	0.05	0.02	0.34	0.2	− 0.04	0.6	0.02	0.7	0.2	0.26	n/a	n/a
MCP1	0.009	0.04	0.20	0.2	− 0.29	0.4	0.26	0.8	0.8	0.06	0.7	0.09
TARC	0.4	0.2	0.19	0.9	− 0.02	0.4	0.11	0.2	0.6	− 0.13	0.2	0.32
MDC	*0.0006*	0.2	0.19	*0.003*	− 0.22	0.6	− 0.05	0.3	0.9	− 0.02	0.2	− 0.29
MIP1a	0.06	0.4	0.13	0.1	− 0.27	0.6	− 0.11	0.3	0.6	− 0.11	0.8	0.07

All cytokine values are log-transformed except for TNFR1 which was square-root transformed. Younger age group used as reference group

Statistically significant values are indicated by italics

*Coef* coefficient, *PD* parasite density, *AG* age group

^a^By Wilcoxon rank sum test

**Fig. 2:**
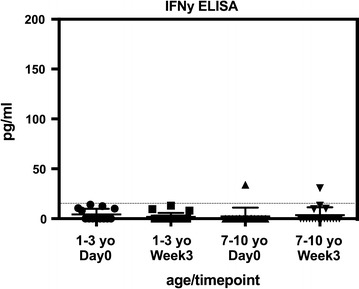
IFNγ responses to malaria across age groups by ELISA. Data are presented as scatter plots across age groups and timepoints. Boxes represent medians and whiskers represent the IQR of each group. The dashed horizontal line marks the lower limit of detection for the assay

Surprisingly, the concentration of IFNγ, as well as the other pro-inflammatory cytokines IL12p70 and IL1β, were below the limit of detection for all samples. Because this lack of detectable IFNγ was in conflict with some previous reports [16, 20–22], a highly sensitive IFNγ-specific ELISA was performed. This more sensitive assay confirmed that plasma IFNγ levels were beneath the limit of detection in all but two samples (Fig. 2), both of which were only minimally higher than the lower limit of the assay. In summary, older children in this highly endemic setting appear to have a blunted cytokine response to acute malaria, with no significant elevation of classic pro-inflammatory Th1 cytokines during acute symptomatic episodes as compared to convalescence, and a notable absence of IFNγ in peripheral blood plasma for both age groups during acute malaria and convalescence.

### Regulatory cytokines including IL10 are markedly higher during acute malaria in young children

Because regulatory responses are important for limiting the pathology associated with malaria infection, we also quantified concentrations of IL10, sTNFRI, IL4, IL5, IL7, IL17 and IL23. As with the pro-inflammatory cytokine levels discussed above, levels of the regulatory cytokines IL10 and sTNFRI were markedly higher in young children compared to older children during acute malaria. IL10 levels were significantly higher in both age groups at day 0 as compared with convalescence (Fig. 1b). However, day 0 levels in the younger age group were tenfold higher than those of older children at this timepoint (mean of 609 pg/ml for ages 1–3; mean of 60 pg/ml for age 7–10). IL10 is produced by myeloid-derived cells and most lymphocyte subsets, and is thought to control immunopathology and symptoms by limiting cellular immune responses [14, 23].

The soluble TNF receptor TNFRI was also elevated at day 0 in both age groups, with significantly higher concentrations in the younger age group (Fig. 1b). sTNFRI plays a complex role in regulating extracellular TNF availability, with the capacity to stabilize bioactive TNF when it is present in low concentrations or, instead, neutralize TNF when the cytokine is present in excess [24]. Little is known about the role of sTNFRI during malaria infection.

**Fig. 3:**
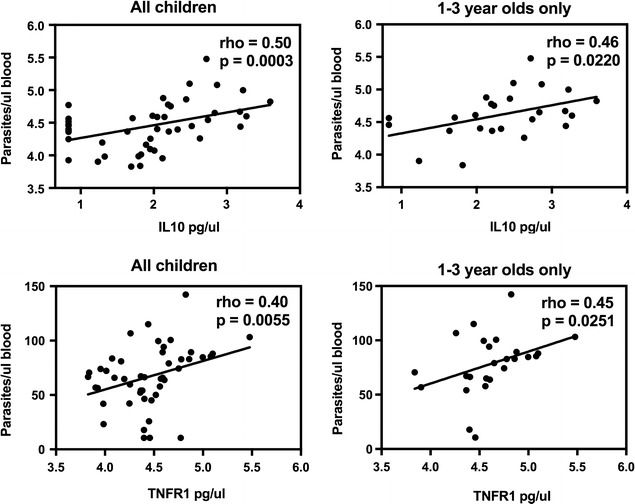
Association between regulatory cytokines and parasitaemia. Spearman’s rank correlation was used to detect associations between plasma levels of IL10 and sTNFRI and parasite density. Parasite density and IL10 concentrations were log transformed, sTNFRI concentrations were square root transformed

Both IL10 and sTNFR1 levels at day 0 were positively associated with individual levels of parasitaemia in univariate analysis (p = 0.0003 and 0.006 respectively). This relationship was significant both in the cohort as a whole and when limiting the analysis to the younger age group (Fig. 3, Table 2), suggesting that the relationship is not simply due to the decline in parasitaemia with age. In multivariate analysis, only age was the significant predictor for both cytokines; however, the interaction between these two predictors was significant, indicating that the relationship between parasitaemia and cytokine concentrations changes with age (Table 2). A positive correlation between IL10 and parasitaemia has been described previously [19, 20, 25, 26]; however, the positive relationship between sTNFRI and parasitaemia is novel.

The Th2 cytokine IL4, while present at detectable levels in many children, showed no relationship with age or time after infection. IL17, IL23, IL5 and IL7 were undetectable in all samples.

### Pro-inflammatory/regulatory ratios are higher in older children

**Fig. 4:**
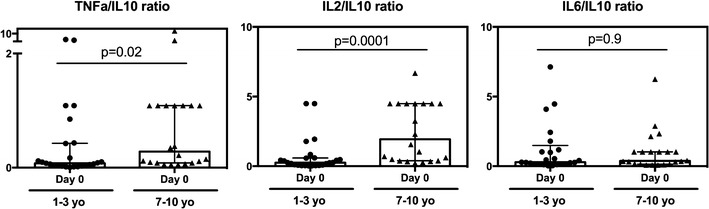
Pro-inflammatory/regulatory cytokine ratios across age groups. Data are presented as scatter plots across age groups and timepoints. Boxes represent medians and whiskers represent the IQR of each group. Significant differences across age groups were assessed by Wilcoxon rank sum

In order to determine whether the diminished regulatory responses seen in older children could be explained by proportionally diminished pro-inflammatory responses in this age group, the relative ratios of TNF, IL2 and IL6 to IL10 at day 0 were compared between younger and older children. For both TNF and IL2, but not IL6, older children exhibited higher ratios of these pro-inflammatory cytokines to IL10 (Fig. 4), indicating that reduced IL10 among older children may not be entirely explained by a reduced need for control of the inflammatory response.

### Th1 plasma chemokines during acute malaria infection are elevated in younger children

**Fig. 5:**
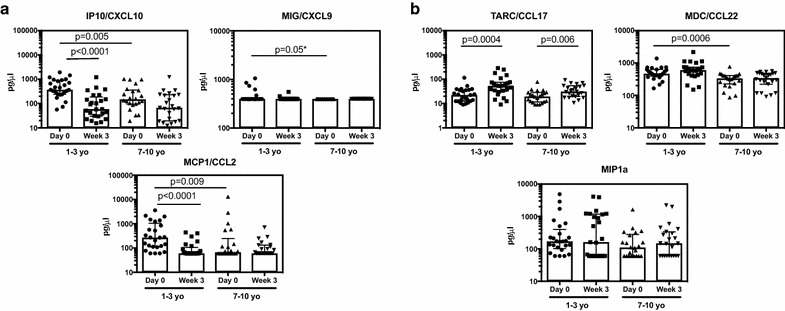
Plasma inflammatory (**a**) and regulatory (**b**) chemokine responses to malaria across age groups. Data are presented as scatter plots across age groups and timepoints. Boxes represent medians and whiskers represent the IQR of each group. Significant differences across age groups were assessed by Wilcoxon rank sum and the Wilcoxon sign-rank test was used for pairwise comparisons between timepoints. Only P values < 0.05 are shown. *Denotes p-values that are not statistically significant (P > 0.0125) after Bonferroni multiple comparisons testing. Samples below the limit of detection are shown at the lower limit of detection. Only chemokines for which > 7% of samples were above the limit of detection appear in the figure

In addition to cytokines, concentrations of several key chemokines were measured. These molecules are crucial regulators of leukocyte trafficking; increased levels of numerous chemokines have been observed in severe malaria [27]. Several chemokines that bind CXCR3, a receptor associated with Th1 CD4+ T cells, were elevated during acute infection in younger but not older children. These included interferon-gamma inducible protein 10 (IP10), also known as CXCL10, Monokine induced by gamma-interferon (MIG)/CXCL9, and Monocyte chemotactic protein 1 (MCP1)/CCL2 (Fig. 5a).

Parasite density trended toward being a significant predictor for these chemokines in univariate analysis, but only age remained significant in multivariate analysis. There were no significant interactions between age and parasite density, and stratifying by age group to look at the influence of parasite density on chemokine concentrations did not reveal any further relationships. (Table 2). Thus, similar to pro-inflammatory cytokine production, younger children are more likely to respond with higher levels of the pro-inflammatory chemokines IP10, MIG and MCP1 during an acute malaria episode, regardless of parasite density at time of infection.

### The Th2 chemokine CCL17 is elevated during convalescence, particularly among younger children

Plasma concentrations of chemokines known to bind CCR4, a receptor associated with Th2 CD4+ T cells, exhibited a different pattern from that described above for CXCR3-binding chemokines. These CCR4-binding chemokines include thymus activation regulated chemokine (TARC)/CCL17, human macrophage derived chemokine (MDC)/CCL22, and macrophage inflammatory protein (MIP1α). TARC concentrations were significantly elevated during convalescence as compared to day 0 in both younger and older children suffering an acute malaria episode (Fig. 5b).

MDC was elevated at both day 0 and week 3 in younger children as compared to the older age group; however, there were no significant differences between timepoints for either age group (Fig. 5b), though concentrations did trend upwards at week 3 in younger children. MIP1α showed no association with age group or time point. Additionally, there were no associations between parasite density and concentrations of these chemokines at any individual time point (Table 2). Thus, despite the Th2-skewing of infant immune cells, chemokines that may have a Th2 bias do not show a strong association with age or with parasite density during acute malaria.

## Discussion

This study sought to determine the effect of age on peripheral blood cytokine and chemokine levels during acute malaria infection in a highly endemic setting. The resulting data indicate that older children respond with diminished levels of the pro-inflammatory cytokines TNF, IL2 and IL6, as well as Th1-biased chemokines when compared to younger children at the same stage of infection. Moreover, older children had strikingly lower plasma concentrations of the regulatory cytokines IL10 and sTNFRI than younger children. Together these data suggest that there is a profound blunting of the cytokine response to malaria among older children residing in endemic settings, which may be due to repeated malaria exposure, to intrinsic age-based differences in the immune response, or to a combination of both factors.

These observations are consistent with the idea that repeated malaria may drive the host towards a disease tolerance state in order to reduce the negative impacts of infection-related pathology [28]. While the exact nature of malaria-induced tolerance requires further investigation, several studies of both innate [29–31] and adaptive [32, 33] cell populations indicate that exhaustion and immunoregulatory pathways play a part. A recent study by Ademoule et al. found that pro-inflammatory cytokine responses during acute malaria, including TNF, IFNγ, IL1β, IL2, IL8, IL6, IL12 and GM-CSF, were decreased in areas with high transmission intensity, lending support to the idea that disease tolerance driven by sustained exposure is reflected by plasma cytokine concentration. Interestingly, TNF, IFNγ, IL6 and IL10 correlated with parasitaemia levels in low transmission areas but not high transmission areas in this study [26]. This last observation is supported by an older study, in which Moncunill et al. found a correlation between IL6, IL10, and parasite density in previously naïve travellers and European migrants, but not semi-immune adults living in a malaria endemic area [21]. There is also a line of evidence suggesting that disease tolerance may involve the generation of neutralizing antibodies to various malarial toxins, including hemozoin [34] and Glycosylphosphatidylinositol anchors [35], binding of which reduces the pyrogenic and cytokine-stimulating capacity of these compounds [36, 37] while leaving parasite replication unaffected. Murine models suggest that tolerance to malaria may in fact come at the price of impairing the host’s ability to limit parasite replication [38], and indeed, amongst human cohorts, asymptomatic infection among children does occur with significant parasitaemia in high transmission areas [26, 39–41]. Thus, dampened cytokine responses may serve to protect individuals from symptomatic disease while at the same time interfering with their ability to prevent re-infection.

Of note, the striking difference in malaria incidence between age groups following sampling, evident in Table 1, may directly reflect the active development of clinical immunity or disease tolerance in older children. Alternatively, this may be an artifact of our small cohort. Amongst the much larger number of children enrolled at this site in the parent study, asymptomatic parasitaemia becomes prevalent around the age of 5 [42], thus, enrollment of children suffering from clinical malaria at the later age of 7–10 may have selected individuals on the verge of developing immunity or tolerance.

The main limitation of this study is its inability to uncouple the influence of prior malaria exposure from that of biological age in this cohort of children. Differences in cytokine production may reflect either the fact that the younger children have sustained less prior exposure to malaria than older children, or instead, intrinsic age-related differences in immune function. Several prior studies support the notion that biological age profoundly influences the development of immunity to malaria. In an elegant study of Javanese transmigrant families emigrating together from an area of little to no malaria transmission to an area of high malaria transmission, adults were found to develop immunity to *P. falciparum* much more rapidly than children [3, 4]; however, infants and young children were much less likely than adults to develop severe malaria requiring medical evacuation [3]. These clinical observations are consistent with the finding that young children mounted an exuberant anti-inflammatory IL10 response, which might limit the inflammatory pathology observed in adults during a first episode of malaria. The increased concentration of IL10 in younger children may reflect the observation that infant monocytes and dendritic cells have been shown to produce less IFNγ, IL12, IL18, and IFNα after stimulation with TLR ligands in vitro, and instead produce more IL10, IL17, IL6, and IL23 than equivalent adults cells [5–7]. This propensity for an enhanced regulatory response may delay the development of robust T cell memory and antibody formation in very young children. A better understanding of how infant immune cells respond differently to infectious pathogens will be crucially important for the development of effective vaccines for children living in endemic areas.

The complete lack of detectable IFNγ or IL12p70, both in younger and in older children, was somewhat surprising. These findings are at odds with some prior descriptions of plasma cytokines during acute infection [16, 20–22], though it is important to note that these studies examined subjects who were previously malaria-naïve or suffering from severe malaria. In subjects who are routinely exposed to malaria infection, the pro-inflammatory response may be diminished or quickly controlled by regulatory mechanisms. This effect may be particularly exaggerated in this study area, where transmission is especially intense. In fact, a recent study of Nigerian pre-school aged children failed to find detectable levels of IFNγ in plasma during acute infection as well [25]. The idea that IFNγ and IL12p70 levels are diminished in frequently-exposed individuals is supported by Ademolue et al., described above, where plasma concentrations of both cytokines were reduced amongst individuals from high transmission areas [26], and by previous work studying malaria-naïve travellers, who exhibit a higher inflammatory response during their first malaria episode as compared with previously exposed and semi-immune individuals [20, 21]. The response of previously naïve individuals in these cohorts included higher plasma concentrations of IFNγ and IL12p70. Both IL2 and IFNγ plasma concentrations were inversely correlated with time since immigration for individuals that had relocated to Europe [20, 21]. Additionally, even in controlled human malaria infection of naive study subjects, a subset of those infected exhibited little to no increase in plasma IFNγ [16], indicating a predisposition towards tolerance (i.e. fewer symptoms but poor parasite control). We cannot completely exclude the possibility, however, that the commercial Luminex and ELISA kits used in this study may have a reduced sensitivity compared to those used in prior reports, and that this may contribute to the lack of detectable IFNγ and IL12p70.

This study had additional limitations, including the inability to record the exact timing of infection, although all samples were obtained within 48 h of the onset of symptoms. Thus, when comparing these data to cytokine concentrations measured during controlled human malaria infection, the timing of sample collection in the present study was less uniform and individual measurements more variable. Helminth co-infection has been shown to impact cytokine responses to *P. falciparum* infection, in some circumstances biasing the immune environment towards a Th2-driven response [43, 44]. As co-infection may be more common in school-aged children [45], this may have confounded our observed age-related differences. Finally, because nearly all children living in Nagongera sustain heavy exposure to *P. falciparum*-infected mosquitoes, the cytokine responses of children from high- and low-transmission settings could not be compared.

## Conclusions

Together, these findings provide new evidence that despite age-related differences in immune responses, young children respond with a largely pro-inflammatory Th1-type cytokine and chemokine response, similar to naïve travellers; however, they also show increased levels of the regulatory cytokines IL10 and sTNFRI, which may limit their ability to develop robust immunity to subsequent malaria infections. This inflammatory response is dampened in older children, suggesting both differences in age-related immune function and the development of partial clinical tolerance.
